# Case report: A novel occurrence of persistent left cranial vena cava coexisting with polycystic kidney disease in a cat

**DOI:** 10.3389/fvets.2023.1268493

**Published:** 2023-10-05

**Authors:** Ye-Eun Cha, Woong-Bin Ro, Seung-Ji Song, Mi-Kyung Lee, Min-Hee Kang, Hee-Myung Park

**Affiliations:** ^1^Laboratory of Veterinary Internal Medicine, College of Veterinary Medicine, Konkuk University, Seoul, Republic of Korea; ^2^Department of Veterinary Emergency and Critical Care Medicine, College of Veterinary Medicine, Chonnam National University, Gwangju, Republic of Korea; ^3^Cat Vet Animal Hospital, Seongnam-si, Gyeonggi-do, Republic of Korea; ^4^Department of Bio-animal Care, Jangan University, Hwaseong-si, Gyeonggi-do, Republic of Korea

**Keywords:** feline, coronary sinus, persistent left cranial vena cava, saline contrast echocardiography, polycystic kidney disease, chronic kidney disease

## Abstract

A 7-year-old castrated male Munchkin cat was presented with anorexia. This cat had been diagnosed with chronic kidney disease due to polycystic kidney disease. Tachycardia with a systolic murmur (grade III/VI) was auscultated and for further diagnosis, echocardiography was performed. Based on echocardiography, persistent left cranial vena cava (PLCVC) was suspected due to enlargement of the coronary sinus and confirmed by saline contrast echocardiography. The dilated coronary sinus compressed the left atrium, and left ventricular hypertrophy with the systolic anterior motion of the mitral valve, aortic regurgitation, and mitral regurgitation were identified. After medical management using atenolol, left atrial function and other hemodynamics of the heart were improved, including the disappearance of regurgitation and normalization of left ventricular wall thickness. This case report describes the echocardiographic characteristics, diagnostic procedures, and disease progression in a cat with PLCVC after medical management using atenolol. Additionally, this is the first report of a cat with PLCVC, coexisting with polycystic kidney disease.

## Introduction

Persistent left cranial vena cava (PLCVC) is a congenital vascular anomaly that occurs when part of the left cardinal vein does not regress and becomes a remnant vessel (PLCVC) during embryonic development. This remnant vessel drains blood from the left side of the head, neck, and forelimb into the right atrium, through the coronary sinus (1–4). In humans, persistent left superior vena cava (PLSVC) is used as an alternative term for PLCVC. PLSVC is the most frequently identified congenital thoracic vascular anomaly in humans, with a prevalence ranging from 0.2 to 3% in the general population, increasing to 1.3 to 11% in individuals with congenital heart disease (3, 5). It has also been reported in several animal species, including dogs, with relatively few occurrences in cats (1, 6–8). In dogs, the prevalence of incidentally detected asymptomatic PLCVC was reported to be 2.6% in a study that used thoracic computed tomography (CT) examination for reasons unrelated to cardiac anomalies (9). However, its prevalence has not been reported in cats.

PLCVC has been reported to occur along with cardiovascular anomalies, although it may also occur independently (1, 4, 6–8, 10). If a PLCVC persists without other cardiac anomalies, it is mostly asymptomatic and may be identified incidentally (1, 2, 6). However, PLCVC can be clinically significant because a dilated coronary sinus (CS) caused by PLCVC can lead to a variety of cardiovascular problems, such as compression of the left atrium (LA), reduced cardiac output, increased risk of thromboembolism, and arrhythmias (3, 6, 7). It can also complicate procedures such as central venous catheterization and surgical interventions (3, 7).

Polycystic kidney disease (PKD) is an inheritable disorder characterized by the gradual formation of fluid-filled cysts in the kidneys, and in certain instances, in additional organs, such as the liver and pancreas (11). PKD is recognized as the most common hereditary disorder in cats, occurring in approximately 6% of the overall feline population with a notably higher prevalence observed in Persian breeds (12). It has been elucidated that the PKD1 gene or other genes within this locus contribute to the pathogenesis of feline PKD (13, 14). PKD is characterized by the progressive diversification of the size and number of cysts over time, eventually leading to chronic renal failure (14). However, the severity and progression of the disease vary among individual animals with no definitive cure. Advancements in genetic testing and diagnostic imaging techniques have made early detection and continuous monitoring of feline PKD feasible (14).

This case report describes the echocardiographic features, diagnostic process, and disease progression in a cat with PLCVC after medical management using atenolol. Additionally, this case demonstrates a novel occurrence of PLCVC coexisting with PKD in a cat.

## Case description

### Case presentation and diagnostic investigations

A 7-year-old castrated male, Munchkin cat weighing 3.8 kg was admitted for a check-up of previously diagnosed chronic kidney disease (CKD) induced by PKD. The cat had been diagnosed with PKD at a young age. During regular check-ups, CKD and hypertension (blood pressure, 175 mmHg) were diagnosed 1 year prior to the current presentation. The renal panels and blood pressure were monitored monthly. The cat had been administered renal supplements (oral spherical carbon absorbent [Renamezin^®^], symbiotics [Renal-advanced^®^], and omega-3 fatty acids [Duomega^®^]), intermittent subcutaneous fluids and amlodipine (0.8 mg/cat, *per os* [PO], q24h, Norvasc^®^). Because the cat had been anorexic for several days before the presentation, an overall examination was performed.

On physical examination, tachycardia (220 bpm) and a systolic heart murmur (grade III/VI) were noted, and systolic arterial blood pressure was normotensive (130 mmHg; Vmed Vet-Dop2™, Vmed Technology, Mill Creek, WA, United States). Complete blood counts (Procyte One Hematology Analyzer™, IDEXX Laboratories Inc., Westbrook, ME, United States) and serum chemistry profiles (Catalyst One Chemistry Analyzer™, IDEXX Laboratories Inc., Westbrook, ME, United States) revealed mild leukocytosis (27, 000/L; reference interval [RI] 5, 500–19, 500/L), increased blood urea nitrogen (41 mg/dL; RI 16–36 mg/dL), creatinine (3.9 mg/dL; RI 0.8–2.4 mg/dL), and symmetric dimethylarginine (19 ug/dL; RI ≤ 14 ug/dL), with normal total thyroxine (2.7 uL/dL; RI 0.8–4.7 uL/dL) and free thyroxine (1.8 ng/dL; RI 0.7–2.6 ng/dL) levels. Results of hepatic enzymes and electrolytes were within normal limits. Urinalysis revealed decreased urine concentration with a urinary specific gravity of 1.011 (RI > 1.035). However, no overt proteinuria was noted.

**Figure 1:**
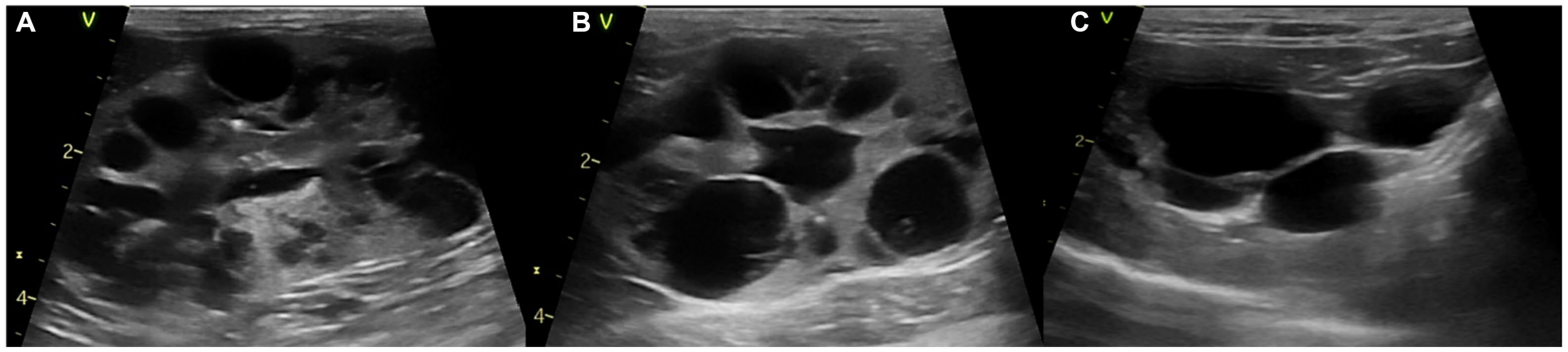
Abdominal ultrasound images of a polycystic kidney disease. **(A)** Left and **(B)** right kidney with polycystic structures of more than 10 cysts. **(C)** Liver with multiple cysts.

As this cat was diagnosed with PKD, abdominal ultrasound showed cysts with variable sizes in both kidneys. Cysts of varying sizes occupied most of the renal parenchyma and renal calculi were also observed in both kidneys (Figure 1). Furthermore, cysts were noted in the liver and bile duct dilation was observed, which increased in size and number compared to the previous examination 6 months before.

A heart murmur was auscultated during the cardiac examination. Thoracic radiography revealed mild cardiomegaly and a mild broncho-interstitial lung pattern. Electrocardiography (ECG) revealed sinus tachycardia with no other remarkable findings (Cardiofax S ECG-1250™, Nihon Kohden Corporation, Toyko, Japan). Two-dimensional echocardiography (Vivid™ T9 Ultrasound System, GE Healthcare, Milwaukee, WI, United States) exhibited left ventricular posterior wall (LVPW) thickening (6.4 mm) with mild left atrial enlargement (ratio of left atrial to aortic root diameter [LA/Ao] = 1.55) and systolic anterior motion (SAM) of the mitral valve in the M-mode. Mild mitral regurgitation (MR) with a maximum velocity of 3.92 m/s and aortic regurgitation (AR) with a maximal velocity of 1.28 m/s due to dynamic left ventricular outflow tract obstruction (DLVOTO) were observed on color and continuous-wave Doppler images. Additionally, the maximal velocity of the left ventricular outflow tract (LVOT) was 4.5 m/s, which was reached at end-systole. The left ventricular contractility was normal to increased (fractional shortening [FS] = 58.09%). The trans-mitral flow velocity on pulsed-wave Doppler imaging was 1.22 m/s with the fusion of early and late diastolic waves due to the fast heart rate (229 bpm), and the mitral annular tissue velocity on tissue Doppler imaging was 0.08 m/s with the fusion of early and late waves.

**Figure 2:**
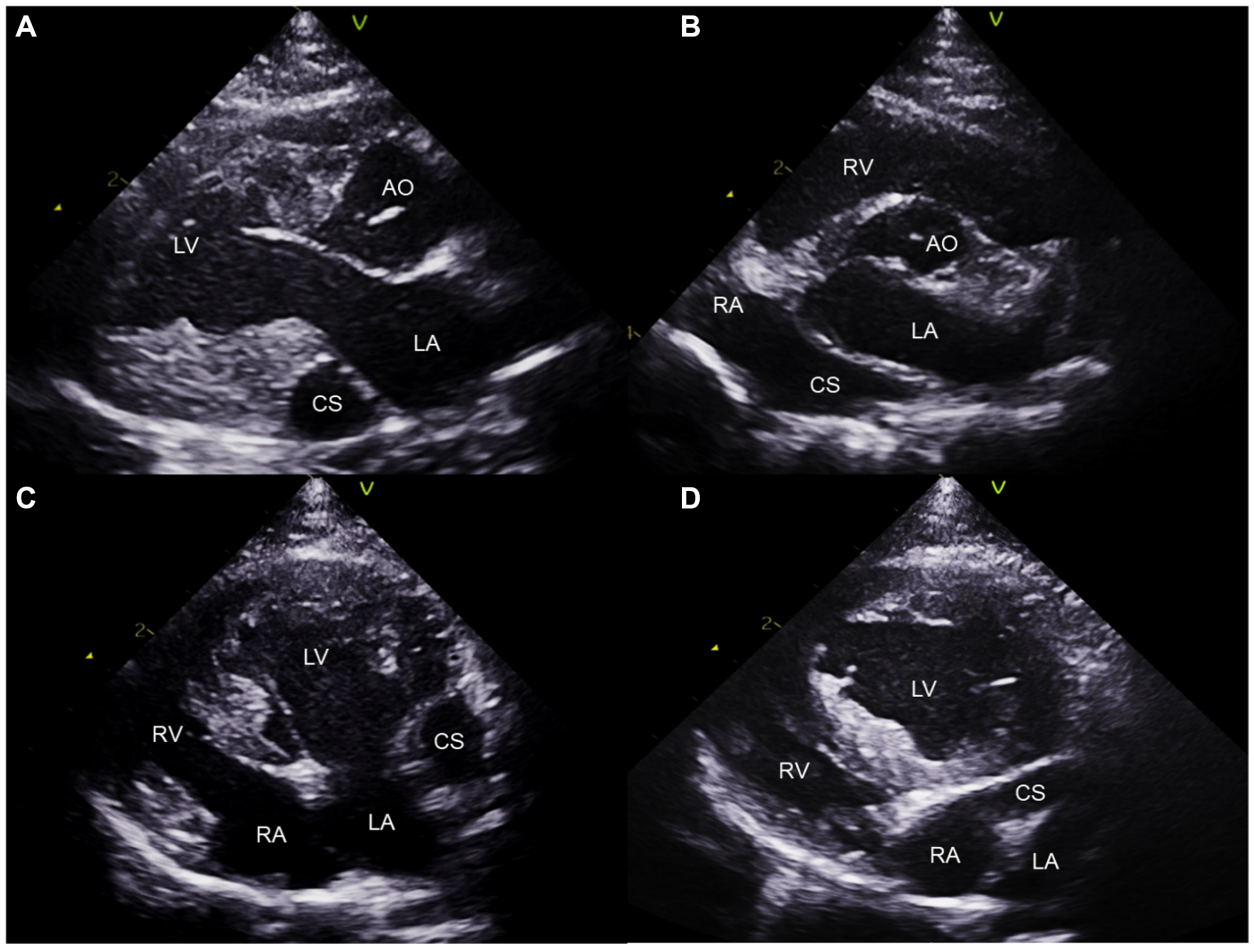
Two-dimensional transthoracic echocardiogram images obtained from the right parasternal long axis **(A)**, right parasternal short axis **(B)**, left apical four-chambers **(C)**, and oblique left apical four-chambers **(D)** views. The dilated coronary sinus is visualized at the level of the LA wall **(A–C)**. Note the continuity of the CS and RA **(B,D)**. CS, coronary sinus; LA, left atrium; LV, left ventricle; RA; right atrium; RV, right ventricle.

Additionally, a round anechoic structure was observed in both the right parasternal and left apical views near the LA (Figure 2). This structure is a dilated CS, located in the left atrioventricular groove, which is not typically observed under normal circumstances. The presence of a cardiovascular anomaly including PLCVC, coronary atrioventricular fistula, and anomalous pulmonary or hepatic venous return was suspected (15), because there was no evidence of elevated right-sided filling pressure, which could contribute to CS enlargement.

**Figure 3:**
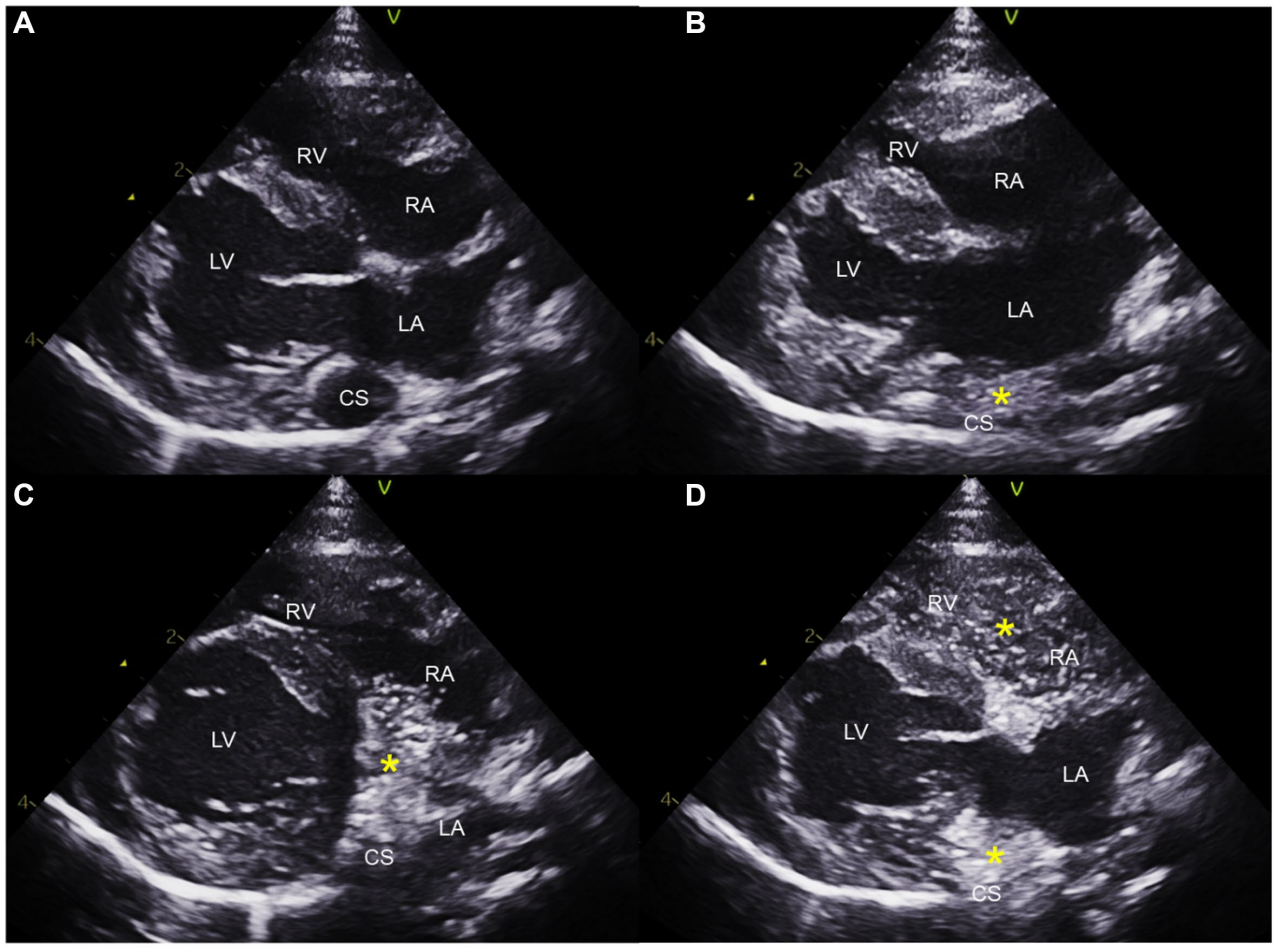
Saline contrast echocardiogram images obtained from the right parasternal long axis view. Note the serial appearance of the microbubbles in the CS and RA injected from the left cephalic vein. Right parasternal long axis view before injection **(A)**, with microbubbles (*) visualized in the CS first **(B)**, with microbubbles moving from CS to RA **(C)**, and with microbubbles in CS, RA, and RV **(D)**. CS, coronary sinus; LA, left atrium; LV, left ventricle; RA, right atrium; RV, right ventricle.

**Figure 4:**
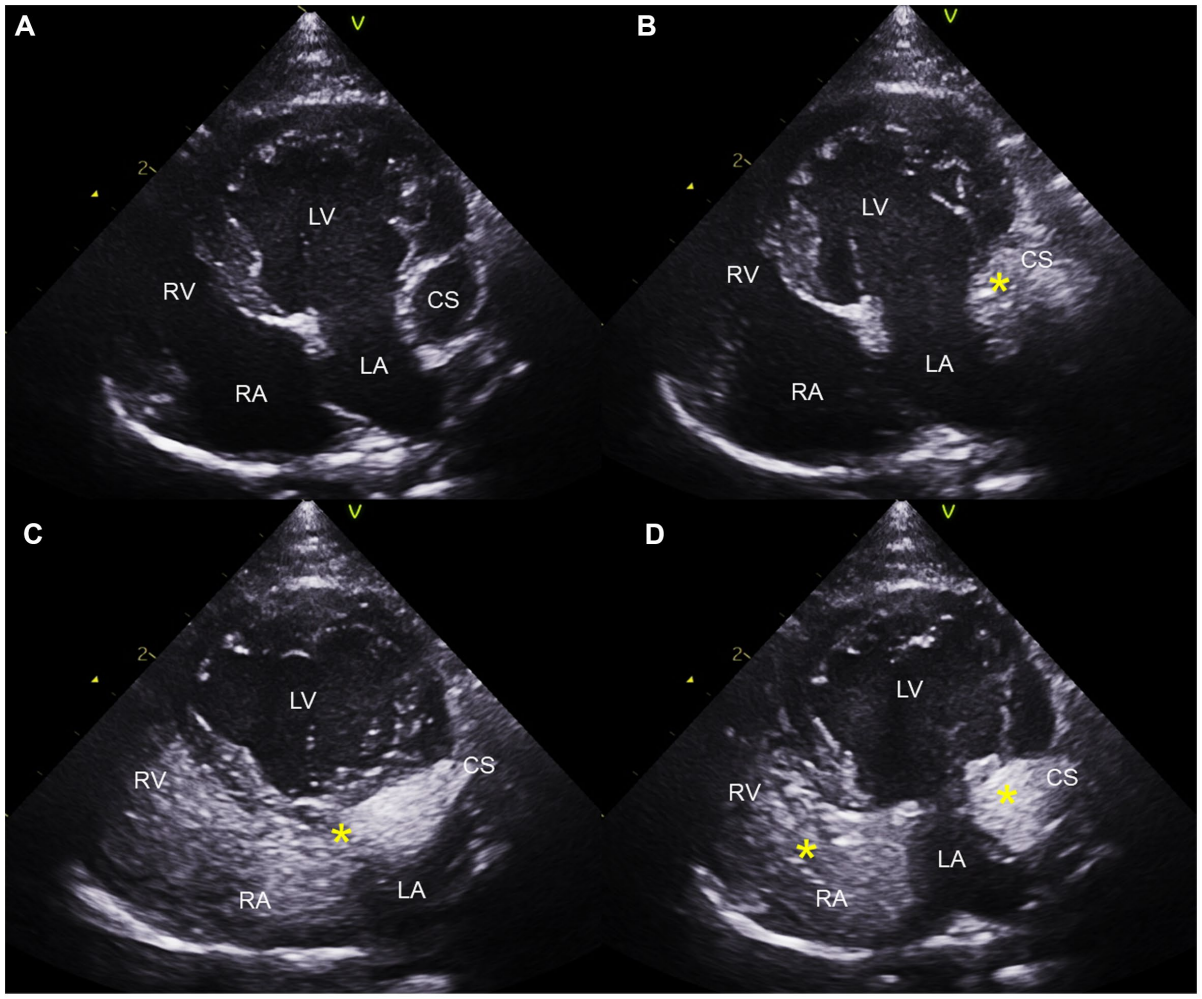
Saline contrast echocardiogram images obtained from the left apical four-chambers view. Note the serial appearance of the microbubbles in the CS and RA injected from the left cephalic vein. Left apical four-chambers view before injection **(A)**, with microbubbles (*) visualized in the CS first **(B)**, with microbubbles moving from CS to RA **(C)**, and with microbubbles in CS, RA, and RV **(D)**. CS, coronary sinus; LA, left atrium; LV, left ventricle; RA, right atrium; RV, right ventricle.

Saline contrast echocardiography was performed to diagnose PLCVC. An agitated saline solution was administered to the left cephalic vein of the cat, and microbubbles entering the dilated CS, followed by the right atrium (RA), were identified (Figures 3, 4), which was confirmative of PLCVC (16–18). However, microbubbles were not observed in the LA, which excluded the unroofed CS. An agitated saline solution was not administered to the right cephalic vein.

Considering the compressive effect of the dilated CS on LA, two-dimensional speckle tracking echocardiography (2D-STE) was performed to evaluate LA movements and function. Only one peak of longitudinal strain was found in the LA because early and late diastolic movements were summated owing to the fast heart rate. The global peak longitudinal strain of the LA (PALS) measured in the left apical four chambers view was 37.7%, which indicated normal to increased movement of the LA with the presence of dilated CS.

Due to the presence of left ventricular hypertrophy, SAM of the mitral valve and suspected diastolic dysfunction with a fast heart rate, the cat was prescribed atenolol (6.25 mg/cat, PO, q24h, Atenol^®^) and clopidogrel (18.75 mg/cat, PO, q24h, Pravic^®^) in addition to previously prescribed amlodipine (0.8 mg/cat, PO, q24h). A follow-up evaluation was performed after 3 weeks. On day 21, the cat’s appetite and activity levels returned to normal. Both heart rate (140 bpm) and blood pressure (130 mmHg) were within the normal range, and echocardiography revealed the absence of SAM, AR, and MR with a reduction in LA size (LA/Ao 1.36). The maximal velocity of LVOT flow was also reduced (1.06 m/s) to the normal range. Additionally, the LVPW thickness decreased to 5.63 mm from the previously measured 6.4 mm on day 0. SAM, AR, and MR were not consistently detected at subsequent visits on day 84. LA size (LA/Ao 1.3) and maximal velocity of the LVOT flow (0.91 m/s) were mildly reduced. Additionally, PALS was increased to 57.5% on day 84, which indicated increased LA movements with the presence of dilated CS and disappearance of DLVOTO and MR. However, the LA strain showed dyssynchronization on day 0 and 84, which indicated myocardial segmental dyskinesia, possibly caused by compression of the dilated CS or independently. However, the systolic arterial blood pressure decreased to 110 mmHg with a heart rate of 160 bpm. Due to the decreased blood pressure, the dose of amlodipine was reduced (0.625 mg/cat, PO, q24h). The cat remained asymptomatic, and vital signs returned to normal. The cat survived for 8 months after the last examination and lost to follow-up.

## Discussion

In humans and dogs, PLCVC is sometimes found incidentally during echocardiography, central venous catheterization, and surgical procedures (2, 6, 8, 18, 19). The available literature on cats primarily focuses on their anatomical characteristic (1, 20–24), with limited reports on its effects on the heart based on echocardiographic features or disease progression. In this case, a diagnosis of PLCVC was established during a cardiac ultrasound, prompted by tachycardia and the presence of cardiac murmurs.

PLCVC occurs when part of the left cranial cardinal vein is not obliterated during embryonic development (1–4). In normal anatomy, only the right cranial vena cava (RCVC) remains and carries blood from the head, neck, and upper extremities to the RA of the heart (2–4). However, in cases of PLCVC, this remnant part of the left cardinal vein is responsible for venous blood return from the left-sided of the head, neck, and forelimb and drains into the CS, and sequentially RA of the heart, independently of the RCVC (3, 6).

As the PLCVC drains into the RA through the CS, it is suspected when the CS is dilated without an increase in pressure in the RA on echocardiography, and the PLCVC can be confirmed through a bubble study using agitated saline in the left cephalic vein (2, 3, 6). The CS is originally connected to the RA; however, when it is also connected to the LA, it results in an R-to-L shunt, which is called an unroofed CS. PLCVC and unroofed CS often occur together and results in hemodynamic changes due to the presence of an R-to-L shunt, which can eventually cause serious clinical symptoms, such as cyanosis (3, 7, 25, 26). In this cat, PLCVC was diagnosed by noticing a dilated CS without an increase in RA pressure and performing an agitated saline contrast echocardiography. The CS was connected to the RA and no bubbles were observed inside the LA; hence, the possibility of an unroofed CS was excluded. In addition, the presence of RCVC can be confirmed by conducting a bubble study from the right peripheral forelimb veins. In the presence of RCVC along with PLCVC, which is called double superior vena cava in humans, microbubbles injected from the right cephalic vein enter the RA. In the absence of RCVC, which is called an isolated PLCVC, microbubbles injected from the right cephalic vein enter the dilated CS before the RA. In this case, saline contrast echocardiography was not performed in the right forelimb, thus, the presence of RCVC could not be confirmed. In our patient, cardiac and vascular malformations other than PLCVC were not observed on echocardiography.

PLCVCs are typically asymptomatic and do not have significant hemodynamic effects (3, 7, 26). However, some reports in humans have suggested that LA pressure may increase due to compression caused by CS enlargement (3, 7). Therefore, in the present case, compression and subsequent pressure elevations in the LA may have occurred as a result of CS dilation. This is particularly significant considering the concurrent presence of the cat’s underlying DLVOTO, which can further contribute to the adverse effects of increased pressure on the LA. Considering these points, the PLCVC in this cat may contribute to advancing the timing of cardiac deformation, particularly with an increase in LA pressure. Furthermore, LA compression and increased LA pressure can increase the risk of developing arrhythmias and thrombosis, which can lead to potentially fatal conditions (3, 7). Arrhythmia was not detected on the ECG performed on this cat.

Additionally, 2D-STE was performed to evaluate the LA function due to compression and pressure increases in the LA (27, 28). The strain value of the LA was 37.7% on day 0 and increased to 57.5% on day 84 after atenolol administration, which indicates decreased LA pressure and improved LA function and movements by slowing the heart rates and relieving the obstruction.

At presentation, the LVPW in this cat was 6.4 mm and did not fall within the normal range. However, the LVPW thickness decreased to 5.63 mm and DLVOTO disappeared after atenolol administration. Therefore, the subtle increase in myocardial thickness in this cat could have been potential implications for pseudohypertrophy, more likely than for hypertrophic cardiomyopathy (HCM). Reduced left ventricle (LV) volume can cause pseudohypertrophy of the LV wall (29). Also, reduced volume in the LV can cause DLVOTO, resulting in secondary LV hypertrophy with tachycardia and reduced cardiac output (30). In addition, dilated CS can exert pressure on the LA and cause a decrease in volume in the LA and subsequently in the LV, resulting in a hypovolemic state of the heart (3). Hypovolemic state may have been caused by other conditions such as CKD (31). Other causes of LV hypertrophy, such as systemic hypertension and hyperthyroidism, were ruled out. If a hypovolemic state persists and hypertension develops to compensate for the reduced cardiac output, kidney function can deteriorate (32).

The cat was diagnosed with PKD in addition to PLCVC. Because PKD in cats is known to be rooted in genetic causes (14), it is likely that these diseases occur together because of genetic defects. According to reports in humans, PLCVC and renal anomalies including PKD, horseshoe kidney, and unilateral absent kidney occur simultaneously (5, 33, 34). In addition, among humans, autosomal-dominant PKD (ADPKD) patients have a higher frequency of congenital heart disease than the general population (35). This is because polycystin proteins encoded by *PKD1* or *PKD2* genes may play crucial roles in cardiac development and function. Cardiovascular diseases are among the most common causes of mortality in human ADPKD patients (35). Although genetic testing for PKD was not conducted for this cat, it was also possible that the cat developed this disease due to underlying genetic factors. Additionally, cats with PKD frequently progress to CKD over time, and the insertion of a central venous catheter may often be necessary for procedures, such as hemodialysis, in cases of end-stage renal disease. Therefore, early evaluation of the presence of congenital abnormalities in the heart and vasculature using methods, such as echocardiography, in cats with PKD may be important for future patient management and prognosis.

This case report had some limitations. First, CT angiography was not performed. CT scans are required to confirm various vascular malformations, including the presence of RCVC and bridging veins. The lack of visualization of PLCVC and vascular structures in this cat is a major limitation, as complex or different types of malformations may occur (3, 9). If patients require interventional therapy with a central venous catheter or surgery, the exact vascular structure of the patient must be determined, and a CT scan is considered essential. However, due to the burden of anesthesia in cats with heart disease, there are clinical limitations to CT evaluation.

Second, the exact etiology of myocardial thickening in this cat could not be definitively determined. At the time of the initial evaluation, the presence of myocardial hypertrophy along with DLVOTO suggested a possibility of HCM. However, decrease in myocardial thickness during monitoring process implied potential for pseudohypertrophy caused by hypovolemic state of the left-sided heart. Otherwise, DLVOTO caused by hypovolemic state may have resulted in secondary hypertrophy of the LV, which resolved after atenolol administration. It is believed that this hypovolemic state could have possibly resulted from other conditions such as CKD (31) as this cat showed low urine specific gravity. In addition, dilated CS could have exerted pressure on LA, deteriorating the situation.

To the best of our knowledge, this is the first report of a cat with PLCVC and concurrent PKD. In this report, we describe a cat with PLCVC, including a diagnostic approach using echocardiography, hemodynamic changes in the left side of the heart, and response to medical treatment using echocardiographic measurements. In cats with PKD, identifying concurrent cardiovascular anomalies, including PLCVC, is important because kidney function is closely related to the cardiovascular hemodynamic changes that may be caused by PLCVC. This case report emphasizes the importance of diagnosing cardiovascular malformations, such as PLCVC, that can coexist with PKD in cats. Recognizing coexisting congenital diseases is crucial for patient management and prognostic evaluation.

## Data availability statement

The original contributions presented in the study are included in the article/supplementary material, further inquiries can be directed to the corresponding author.

## Ethics statement

Ethical approval was not required for the studies involving animals in accordance with the local legislation and institutional requirements because this is a case report of a patient. Written informed consent was obtained from the owners for the participation of their animals in this study.

## Author contributions

Y-EC: Conceptualization, Data curation, Writing – original draft, Writing – review & editing. W-BR: Conceptualization, Data curation, Writing – original draft, Writing – review & editing. S-JS: Data curation, Writing – review & editing. M-KL: Data curation, Writing – review & editing. M-HK: Supervision, Writing – original draft, Writing – review & editing. H-MP: Supervision, Writing – review & editing.

## Figures and Tables

**Table 1:** 

PLCVC	persistent left cranial vena cava
LCVC	left cranial vena cava
PLSVC	persistent left superior vena cava
CS	coronary sinus
LA	left atrium
PKD	polycystic kidney disease
CKD	chronic kidney disease
RI	reference interval
LVPW	left ventricular posterior wall
SAM	systolic anterior motion
MR	mitral regurgitation
AR	aortic regurgitation
DLVOTO	dynamic left ventricular outflow tract obstruction
LVOT	left ventricular outflow tract
2D-STE	two-dimensional speckle tracking echocardiography
PALS	peak longitudinal strain of the left atrium
RA	right atrium
ADPKD	autosomal-dominant polycystic kidney disease
HCM	hypertrophic cardiomyopathy.
